# Isolation of *Cryptococcus gattii* from feline chronic stage lipoid pneumonia

**DOI:** 10.1016/j.mmcr.2019.06.002

**Published:** 2019-06-12

**Authors:** Robert Newman, Megan Schaible

**Affiliations:** Southwest Veterinary Surgical Service, 22595 N Scottsdale Rd #120, Scottsdale, AZ, 85255, USA

**Keywords:** Feline, Pneumonia, Cryptococcus gattii, Pulmonary mass, Lung lobectomy

## Abstract

We hereby report a unique manifestation of feline cryptococcosis in the form of extensively consolidated lipoid pneumonia resulting in an isolated space-occupying lung lobe mass. Lung lobectomy was performed and a diagnosis of severe lipoid pneumonia with intra-lesional *Cryptococcus gattii* was made. The patient was asymptomatic for respiratory disease and no abnormalities of the upper respiratory tract were identified. Anti-fungal therapy was initiated following the diagnosis and the patient recovered without complication.

## 1 Introduction

*Cryptococcus gattii* is a common systemic fungal agent in cats and is a particularly important upper respiratory pathogen in this species [1]. Common clinical signs associated with *C. gattii* in cats include nasal discharge, sneezing, dyspnea and ulcerations or growths affecting the nasal and facial regions. Secondary central nervous system involvement resulting in seizures, mentation deficits and blindness, as well as disseminated disease resulting in dermatological lesions are also well-documented [2]. However, reports of *C. gattii* as a proposed causative agent in consolidated feline lower airway disease (i.e. pneumonia), especially in cats devoid of upper respiratory disease, are lacking. The herein reported case is that of such a circumstance and therefore provides insight into a unique manifestation of the disease. In the present case, an asymptomatic cat was presented to a referral surgical institution for lung lobectomy after incidental discovery of a large and poorly defined lung lobe mass. The facial and upper respiratory anatomy of the patient examined unremarkably. Histopathological assessment and culture of the mass returned a diagnosis of severe lipoid/xanthogranulomatous pneumonia and intra-lesional cryptococcosis. Cryptococcosis was later confirmed serologically.

Lipoid pneumonia results from lipid accumulation within the alveoli and occurs in both endogenous and exogenous forms [3]. Lipoid pneumonia can be idiopathic in origin however pulmonary infiltrative disease or space-occupying masses resulting in excessive surfactant production, such as bronchogenic carcinoma [4] or infection with *Mycobacterium fortuitum* [5], are reported predisposing causes in cats. To the author's knowledge this is the first reported instance of a feline diagnosed with lipoid pneumonia as a proposed result of lower airway *C. gattii* infection.

## 2 Case

**Fig. 1:**
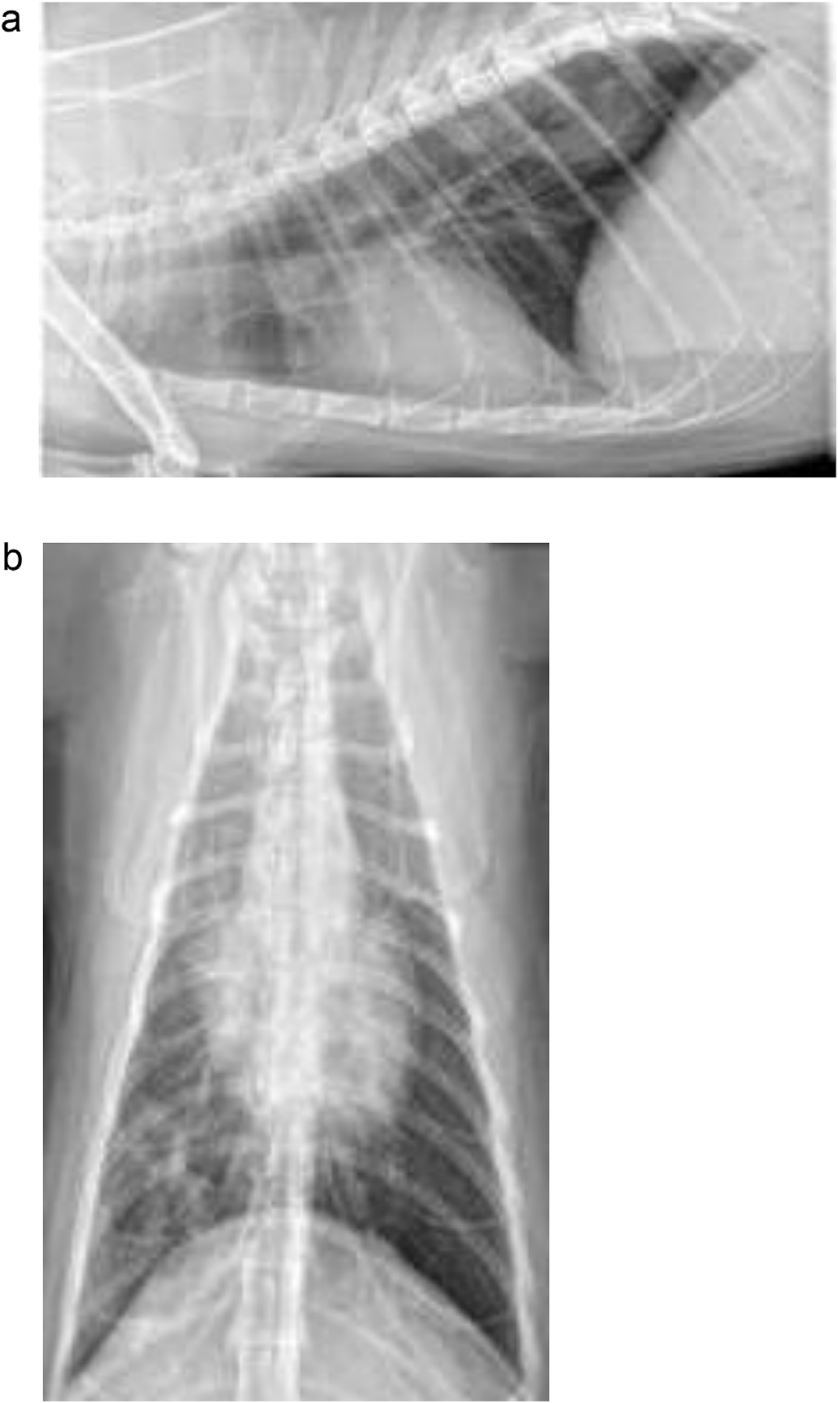
Right lateral and ventro-dorsal thoracic radiographs revealing a consolidated right caudal pulmonary mass-effect.

An 8-year-old neutered male domestic short hair cat weighing 5.0 kg presented to a surgical referral center for right caudal lung lobectomy following diagnosis of a right caudal lung lobe mass on thoracic radiographs (Fig. 1) (day 0). The patient was asymptomatic for respiratory disease and the radiographs were performed by the primary care veterinarian as part of a diagnostic work-up for a newly detected I/VI parasternal systolic heart murmur. The patient had a medical history of chronic crystaluria and had been treated previously for urethral obstruction. The patient had no known travel history.

ECG and echocardiogram were performed by a board certified veterinary cardiologist and revealed no significant abnormal findings. Complete blood count (CBC) and blood chemistry were performed and were within normal limits. Given the patient's geographic location (Arizona, USA) coccidiomycosis (“Valley Fever”) titers were assessed via serology and found to be negative.

**Fig. 2:**
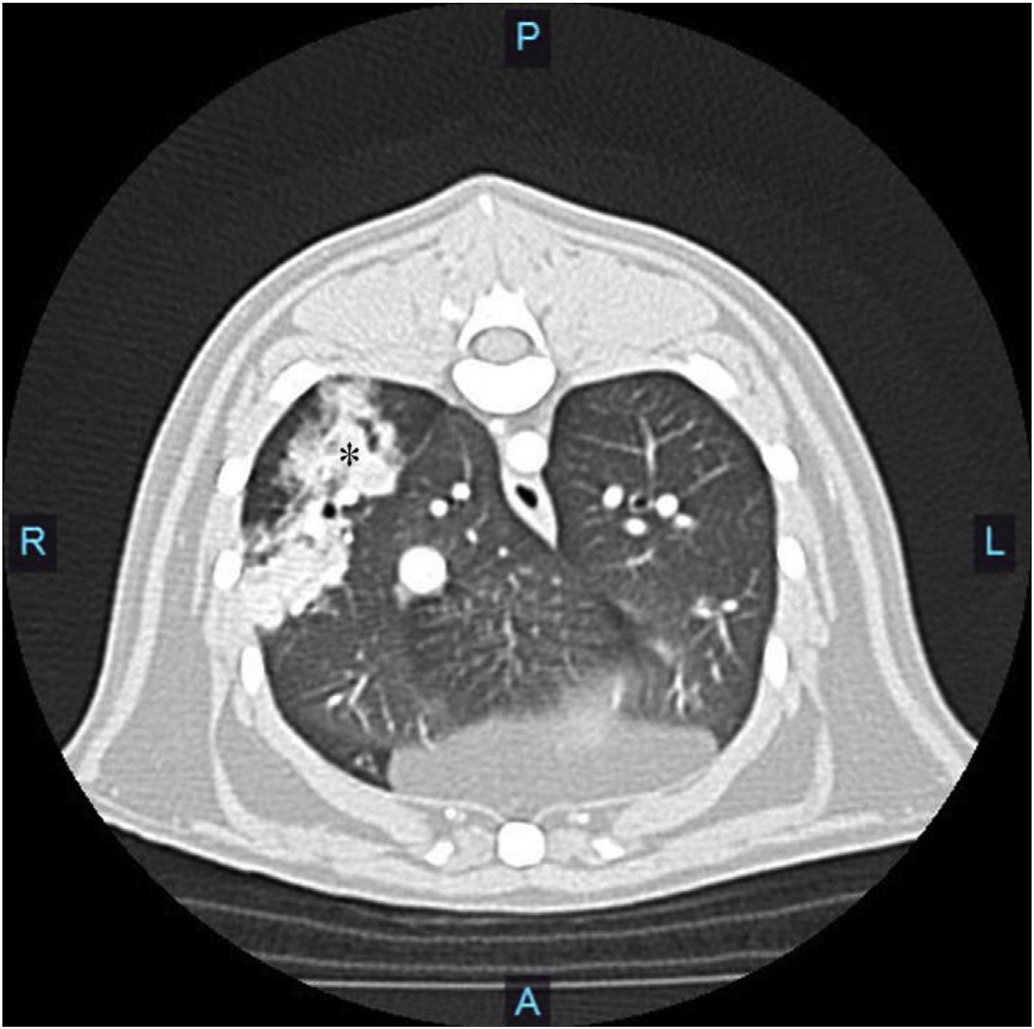
Transverse contrast enhanced 2mm CT slice of the caudal thorax revealing an amorphous, contrast enhancing, airway encompassing, dorso-lateral right caudal lung lobe lesion of an overall length of approximately 5 cm. The lesion is highlighted by an asterisk (*).

**Fig. 3:**
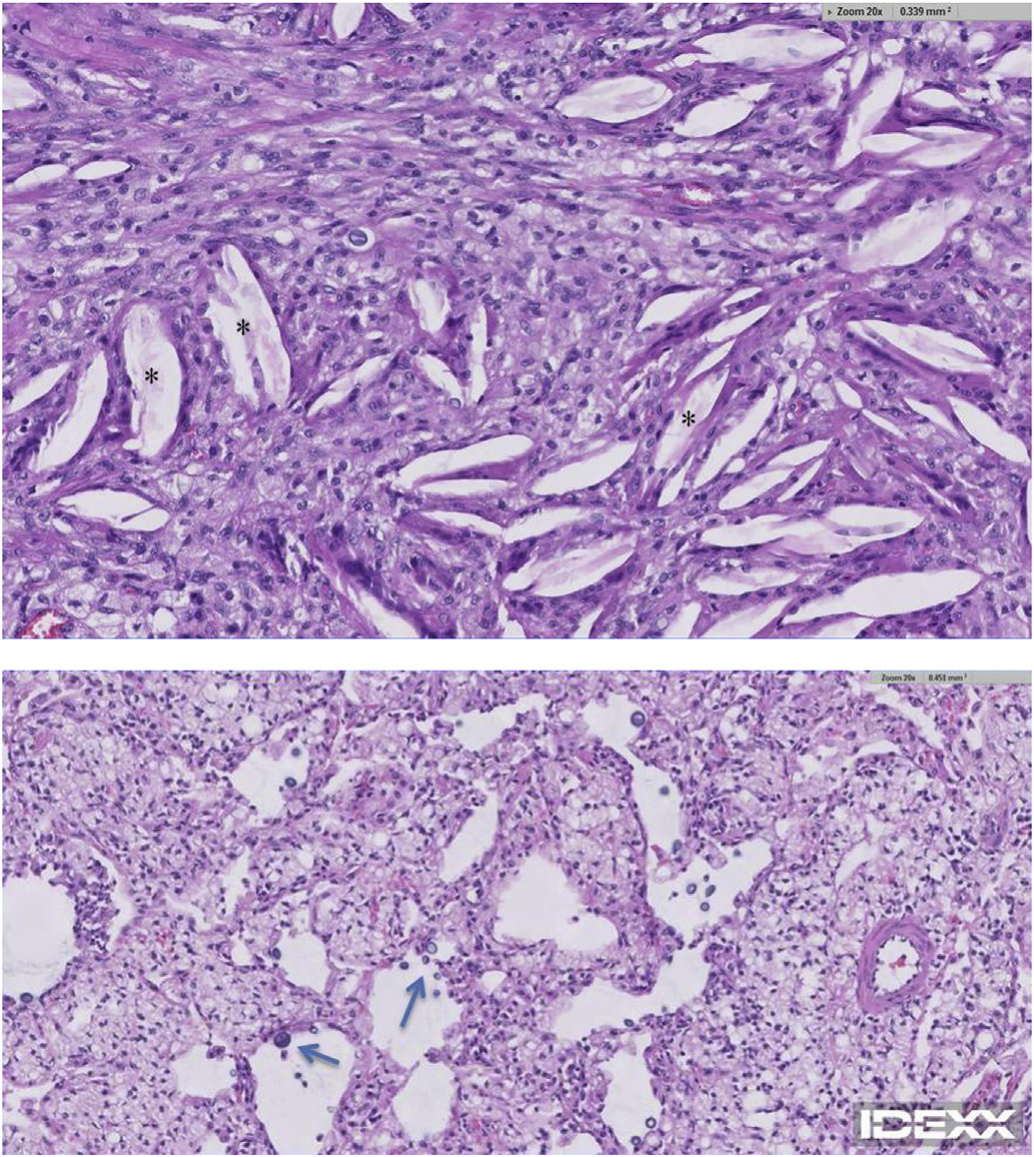
Histopathological sections of removed caudal right lung lobe mass revealing a markedly expanding inflammatory focus consisting of large numbers of epithelioid to foamy macrophages with lesser numbers of multinucleated macrophages, lymphocytes, plasma cells and neutrophils. Marked cholesterol crystal/cleft deposition is noted (asterisks), consistent with chronic stage endogenous or exogenous lipid pneumonia. Scattered intra-lesional organisms consistent with *C. gattii* are present (arrows).

Computed tomography (CT) (Toshiba Aquilion 16) of the thorax was performed and revealed an amorphous contrast enhancing mass in the peripheral right caudal lung lobe (Fig. 2) without evidence of regional lymphadenopathy. Differentials for the mass included a bronchogenic carcinoma or a non-discrete inflammatory or infectious lesion. The patient was taken to surgery and a right caudal lung lobectomy via a right 6th intercostal thoracotomy was performed. The excised lung lobe was submitted for histopathological assessment and microbial culture. A thoracotomy tube was placed and the surgery was concluded without complication. The patient recovered successfully and was discharged from hospital the following day on oral anti-inflammatory (Robenacoxib, 6 mg PO SID), opioid (Buprenorphine, 0.1 mg PO TID) and antibiotic (Amoxicillin-Clavulanic Acid, 62.5 mg PO BID) medications. Three days following surgery, histopathology results were available and revealed a widespread lipoid/xanthogranulomatous pneumonia with scattered intra-lesional organisms consistent with *Cryptococcus spp*. (Fig. 3). No evidence of neoplasia was detected. Five days following surgery, aerobic culture results were available and revealed the isolate *C. gattii.* No organisms were isolated on anaerobic culture.

Following the returned histologic and culture diagnosis, Cryptococcus titers were serologically assessed for confirmation and found to be positive (1:256) (day 10). The cat was started on anti-fungal therapy (Fluconazole 100 mg SID) and has remained free of clinical disease at the time of last follow up (60 days).

## 3 Discussion

Cryptococcosis is a routine differential diagnosis for feline patients presenting with gross lesions affecting the face and/or upper respiratory tract. Colonization of the nasal cavity is particularly well documented in this species [6]. Various reports also exist of *Cryptococcus spp.* isolated from the external ear canal [7], nasopharynx [8] and eyelids [9] of cats as well. However, evidence of the disease affecting the lower airway of cats in apparent isolation appears to be rare. A single report does exist of a cat with an identified pulmonary mass from which intra-lesional Cryptococcus was identified [10]. However, unlike in our case, the infecting cryptococcal species in that patient was *C. neoformans*, not *C. gattii.* Additionally, unlike in our case, the cat in that scenario had presented with acute respiratory clinical signs and pleural effusion. In humans *C. gattii* is already a well-reported causative agent of pneumonia [11]. A similar report to ours of a human patient who presented with an aggressively expanding pulmonary mass secondary to *C. gattii* is available [12].

The diagnosis of intra-lesional *C. gattii* within a pulmonary mass of consolidated lipoid pneumonia for the herein reported patient was unanticipated. The primary differential prior to surgery was a bronchogenic neoplasm, however infectious or inflammatory differentials could not be ruled out. It is important to note that no clinical evidence of upper respiratory or otherwise disseminated or stand-alone cryptococcosis could be appreciated in this patient, either in real time or in retrospect. Previously reported stand-alone or disseminated forms of the disease in cats include cutaneous involvement [13] or that of the central nervous system [14]. It is also interesting that the removed pulmonary mass represented an incidental finding in a cat devoid of any apparent respiratory clinical signs. Noteworthy too is that, despite the localized pulmonary isolation of *C. gattii* in this patient, regional lymphadenopathy was neither appreciated on CT nor during surgery. The geography (Arizona, USA) and lack of significant travel history in this patient is also important to note.

In this report we document a unique manifestation of *C. gattii* in a feline patient. Had cryptococcosis been considered a differential diagnosis for the lung lobe mass initially, serological testing for this infection could have been recommended. Had serological detection of *C. gattii* been achieved initially then medical treatment, rather than surgery, may have been the best initial treatment recommendation. As such, this report provides a justified basis for considering and serologically testing for cryptococcosis prior to surgical excision of isolated lung lobe lesions in feline patients, regardless of geographic location or travel history of the patient. Given the geographical location of the case, such exact consideration was given to the more common regional fungal organism *Coccidioides* for which the patient tested serologically negative. This report adds to the currently growing body of knowledge surrounding cyptococcal infections in cats and confirms the disease as a differential diagnosis for consolidated lung lobe masses in this species. Having occurred in Arizona of the USA, this report also contributes to the growing epidemiological database supporting world-wide emergence of this fungal pathogen [15].

## Conflict of interest

There are none.
